# The Failures of Ethnobotany and Phytomedicine in Delivering Novel Treatments for Snakebite Envenomation

**DOI:** 10.3390/toxins12120774

**Published:** 2020-12-06

**Authors:** Steven A. Trim, Carol M. Trim, Harry F. Williams, Sakthivel Vaiyapuri

**Affiliations:** 1 Venomtech Ltd., Sandwich, Kent CT13 9FE, UK; 2 School of Psychology and Life Sciences, Canterbury Christ Church University, Canterbury CT1 1QU, UK; carol.trim@canterbury.ac.uk; 3 Toxiven Biotech Private Limited, Coimbatore, Tamil Nadu 641042, India; harry@toxiven.com; 4 School of Pharmacy, University of Reading, Reading RG6 6UB, UK

**Keywords:** snakebite, envenomation, ethnobotany, drug discovery, medicinal plants, phytomedicine, traditional treatments

## Abstract

Snakebite envenomation (SBE) is a high-priority, neglected tropical disease. This devastating occupational health hazard disproportionately affects rural farming communities in tropical countries. This is exacerbated by the distribution and densities of venomous snakes, incidence of encounters, and limited access to advanced healthcare, including antivenom. Before the development of antivenom, desperation and spiritual beliefs led patients to experiment with a wide range of traditional treatments. Many of these treatments still survive today, particularly in regions where access to healthcare is limited. Plants are a major source of bioactive molecules, including several lifesaving medications that are widely used to this day. However, much of the research into the use of traditional plant treatments for SBE are limited to preliminary analysis or have focused on techniques used to confirm antibody efficacy that are not suitable for non-antibody-containing treatments. Modern drugs are developed through a robust pharmaceutical drug discovery and development process, which applies as much to SBE as it does to any other disease. This review discusses specifically why research into ethnobotanical practices has failed to identify or develop a novel treatment for SBE and proposes specific approaches that should be considered in this area of research in the future.

## 1. Introduction

Snakebite envenomation (SBE) can be a catastrophic event in a person’s life. Within a fraction of a second, their entire future may change as a result of SBE-induced complications. This leads not only to huge morbidity and mortality [1,2] but to significant socioeconomic ramifications and long-term physiological sequalae and/or psychological trauma [3]. It is therefore not surprising that desperation has led patients to seek any treatment that promises temporary or permanent relief. It was not until 1897 when the first antivenom was produced. Then called antivenin, due to its French origin [4], it was the first truly effective treatment to become available for SBE. It took many decades for antivenom to be produced to treat envenomation from the majority of snake species responsible for SBE. However, antivenom is not 100% effective or totally safe and it is also vitally important to use the right antivenom [5,6,7]. Many improvements are still essential for the effective treatment of SBE. The cost of antivenom is a major factor, limiting accessibility to proper treatment in the impoverished areas where SBE is most common. This forces people into using traditional medicines from locally available traditional healers, who are more accessible and much more affordable than modern medical facilities.

In the thousands of years of human history prior to this ground-breaking antivenom treatment, many strange, and sometimes dangerous, practices were tried in an attempt to save limb and life following SBE. For example, pouring crude leaf extracts from *Ficus religiosa* in the ears of snakebite victims has been reported from the tribes of Uttar Pradesh, Northern India [8]. Mystic approaches are common. Touching the healer, or jolemo, is described in Kenya [9], and mantra (chanting) in Orrissa [10]. Black stones are also commonly discussed as the belief is that the porous nature of the burnt bone (called a black stone) will absorb the venom toxins [11]. Aqueous extracts from peacock feathers and ash are also believed to be effective for snakebites [12], and the application of cow dung after making incisions into the skin that, unsurprisingly, lead to severe infections and subsequently significant complications, including sepsis [13]. Consumption of alcoholic beverages is also a commonly used treatment, which often leads to further complications [11] but may aid the patient’s mental state. In an extraordinary documented case, 96 chickens were applied live, sequentially, with their “anuses stretched”, to remove the venom from a large binocellate cobra bite (presumably *Naja naja*) [14]. Although this was published in 1928, the attending medical officer took this indigenous approach as he had no antivenom and reported it was in vogue in the Ratnagiri district in India. As the patient apparently survived, it is logical to suspect a dry bite in this case. All of these examples serve to highlight the significance of traditional treatment in the management of SBE in rural communities, with many still in use today.

## 2. Medicine or Magic?

When a snakebite patient first presents to a healer or traditional medical practitioner it can be unclear whether envenomation has taken place or not. Moreover, if SBE has occurred, the extent of the envenomation, i.e., the dose of venom injected, may not be known. In the modern hospital setting, the dose of antivenom delivered is vaguely titrated on the basis of the clinical symptoms presented. In the 1928 case where an SBE patient was treated with “chicken”, no symptomatic evidence of systemic envenomation was found [14]. The aims of the traditional healer may well be focused on the perceived “evil” or “magic” of the event without any scientific basis. On the other hand, some traditionally used herbal plants may have some scientifically valid reasons due to the presence of active ingredients to inhibit venom toxins. However, the published literature is widely understood to be biased to those cases where traditional medicine has failed or not completely effective and the patient has then sought medical help when the situation gets worse. There is a lack of documentation of case reports from traditional healers and correlation with dose of envenomation to actually understand if there is any actual benefit. Notably, where benefits were claimed, they were largely in cases of non-venomous or dry bites where no clinical symptoms of SBE were reported. 

## 3. Plant-Derived Medicines for Human Use 

The majority of traditional approaches to SBE use plants from a wide variety of species. This is probably not surprising, as plants often dominate the natural landscape and make up a large part of our diet. Yet, many plants are dangerous to eat, implying they contain compounds with pharmacological activities that have been construed, by some groups, as magic. The presence of such compounds is often a result of a plant’s defence system, producing toxic compounds not only as deterrents against herbivores, but as antimicrobials to prevent the colonisation by bacteria and fungi. Many of these compounds might be from endophytes rather than the plants themselves [15].

Plants are a significant source of licenced drugs, used throughout the world for the treatment of a wide range of diseases. Estimates suggest approximately 25% of licenced drugs come directly from plant compounds [16], although the figure for natural compounds is much higher when we consider the use of their synthetic derivatives [15]. These licensed plant compounds differ from the traditional medicines because they repeatedly produce a significant benefit to the patient, over and above the placebo control, and have passed rigorous safety and efficacy tests. For example, morphine from the poppy (*Papaver somniferum*) has its origin in ethnobotany and its species name implies this was used long before the adoption of the binomial naming system—the Latin *somnifere* translates roughly to “sleeping pill” in English. Plant compounds such as taxol and vincristine have made a huge improvement to the lives of numerous cancer patients [16], and as such, novel production methods for these compounds are being tested [17]. Other examples used in modern medicine include atropine, digoxin, quinine, cannabidiol, and others.

Plant compounds also provide inspiration, are useful starting material, and act as a template for modern drug design and development. Aspirin (acetylsalicylic acid) is often mistakenly believed to be a natural plant compound. In fact, oxidation of salicyl alcohol (the compound found in willow bark) is required to produce salicylic acid, which in the 1850s was chemically acetylated to acetylsalicylic acid [18].

It is hard to overestimate the huge clinical value of plant compounds originating in treating various human diseases. Despite many successful plant-derived drugs, why has ethnobotany and phytomedicine, looking at a vast range of medicinal plants, failed to deliver any novel treatments to SBE? The answer to this critical question lies in the primitive research being undertaken. There is often no effort to use modern approaches to isolate and characterise active pharmaceutical ingredients (APIs) from useful plants, either by applying robust multidisciplinary tools, or by testing them using appropriate methods. Such manuscripts also omit discussion of potential synthesis of such a drug to make it available for use.

## 4. The Ethnobotanical Pharmacopeia Used for SBE

A survey of traditional medicines in Tamil Nadu, India, identified 72 species of plants from 53 families considered useful for SBE [19]. Similarly, 30 species of plants were cited from a survey in rural Kenya, including native and exotic plants that had to be sourced [20]. In South America, *Philodendron* [21] and *Mandevilla* [22] extracts are thought to be beneficial for SBE. Notably, the ingestion of tamarind juice or soap, the application of calcium carbonate, or secretions of plants such as the genus *Calotropis* are also being routinely practiced in rural settings for snakebite victims [23]. Belief in the therapeutic use of plants has conserved plants such as *Acorus calamus, Buchanania lanzan* (stem bark), *Moringa oleifera* (stem, leaves), *Achyranthes aspera, Gynandropsis gynandra,* and *Bombax ceiba*, whose rhizome pastes are thought to act as antidote for SBE and scorpion sting [24].

## 5. Consequences of Using Ineffective Traditional Treatments for SBE

There is a lack of robust research to corroborate the impact of plant-based traditional treatments on SBE-induced complications. Despite this, many rural communities still rely on them as their first line of defence [25]. Misguided attempts to treat the envenomation can lead to severe delays in seeking effective hospital treatment, including antivenom and additional clinical symptomatic support [25]. Frequently, this delay results in additional complications and subsequently adds to the treatment cost, which can impose significant socioeconomic ramifications on the victims or their families.

Since traditional therapies are easily accessible within rural and tribal areas, they are often sought in preference to more distant alternatives. Only when the situation becomes progressively worse is the need to travel to hospital realised. Unfortunately, in many cases, the victim will die before reaching effective care [23,25]. Limited access to healthcare facilities in rural areas is a critical factor that exacerbates SBE-induced deaths and disabilities. Our previous study [23] has also highlighted the lack of sufficient public awareness about snakes and snakebites as being one of the critical factors in causing people to seek traditional treatments and delay essential hospital care. In addition to the worsening of SBE pathology, traditional remedies also bring risks of extra disease, worsening and potentially weakening the patient. Such complications include infection [13]; injury from freezing, burning, cutting, or tourniquets [11]; and even intoxication [26]. Liver toxicity is associated with bajiaolian (*Dysosma pleianthum*), a Chinese medicine used for snakebite [26], and *Arnebia benthamii* used in Kashmir [27]. 

## 6. Issues Associated with the Research on Therapeutic Efficacy of Plant Extracts for SBE

Publications evaluating traditional remedies for treatment of SBE often claim, beyond the data, that these preparations are effective and generally applicable for all snakes, regardless of their toxicity. The authors frequently only demonstrate in vitro data, with crude plant extracts, on neutralisation of one or a few venom enzymes, or in vivo data with poor relevance to SBE clinical situations. These examples frequently include preincubation of the crude preparation with the venom, before injection into test animals [19,21,22] or shortly thereafter [28], thus creating a situation for inhibition unlike any real snakebite. A review in 2012 on herbal approaches to SBE frequently mentions pre-incubation of the venom with the plant extract, which has no clinical relevance to envenomation [24]. Bitter [9,19] and pungent extracts including spices such as pepper [28] are popular. There are also claims that long pepper extract and piperine produce antibodies that are useful against Russell’s viper bites [29]. We were unable to find papers where the active preparation was directly applied to the model snakebite wound in the test animal, which is often the route of administration of such remedies [15,16,19,21]. Oral administration of test compounds does replicate the mode of application but not patient time scales, as these compounds often need to be given by a healer [9,20,30]. In some instances, oral treatments have been published as failing [21], or in others, the timeframe is immediately before the venom challenge [19]. Purified DNase I from *Tricosanthus tricuspidata* extract does show promise in protecting tissue from released DNA nets following *Echis carinatus* envenomation, but this is only one pathological element of the many involved in SBE and hardly a promising lead. Although not specifically reported from *Echis carinatus,* DNase activity is present in a wide range of snake venoms [31], and as such, there seems little benefit in adding additional enzyme to the injury. If it were a truly useful therapeutic, purified DNase I would be widely used and available.

Beliefs in traditional medicine, including ethnobotany, are thought to persist through the frequency of dry bites and bites from non-venomous snakes not being distinguished from SBE events [11,32]. In this way, apparently miraculous recoveries are possible. However, SBE patients often have to turn to modern medicine when traditional methods fail. Many authors attempt to prove that the traditional methods work, rather than investigate if they work in the way they are used on human victims. This approach is useful to understand whether the belief may be sustained through observation of non-lethal snakebite, or if there is a therapeutic utility. 

## 7. Approaches that Should Be Considered in Ethnobotany Research for SBE

Despite the shortfalls in ethnobotany research for the treatment of SBE, the real benefit of studying ethnobotanical attempts is to discover whether there are robust drug-like molecules from these plants that could make for successful treatments. Many plant extracts demonstrate inhibition of snake venom metalloproteases (SVMPs) and yet they have not made an impact clinically, due to the lack of sufficient characterisation of the specific bioactive API(s). SVMPs are responsible for many life-threatening pathologies, predominantly permanent disabilities following viper bites [2]. Some of the matrix metalloprotease inhibitors have demonstrated in vitro neutralisation of SVMPs [33] and in vivo survival of venom challenge when pre-incubated or dosed immediately after [34]. Even though these drugs have reached clinical phase III trial for cancer [35], the data so far in SBE show them to be comparable to many plant preparations as previously discussed. One example of such compounds is piperine, which has completed phase II for dysphagia [36], which is also supported by strong absorption and bioavailability data [37]. 

Whilst there are few randomized, controlled trials for antivenom administration, robust clinical use of specific antivenoms demonstrates their lifesaving efficacy [6,7,11]. However, antivenoms are not without any issues and risks. Type I and III hypersensitivity reactions [6]; poor stability [11]; and a lack of oral bioavailability, necessitating injection, are all problems. Notably, the cost of antivenoms is often financially crippling for poor rural labourers and their families. Thus, there is a desperate need for compounds that can neutralise the major lethal effects of SBE whilst being stable in tropical temperatures, with good oral bioavailability and a robust safety profile. Many ethnobotanical preparations have these properties, so why are they not entering clinical trials? The problem comes from the approach and a lack of basic drug discovery principles. Almost all papers reviewed for this manuscript followed the World Health Organisation (WHO) guidelines for antivenom efficacy testing [38], however, this is purely for antibody therapies and specifically warns against extrapolating such data to indicate clinical efficacy. This method is only appropriate for specific immunoglobulin-based therapies, commonly called antivenom, and does not imply efficacy in the clinical setting of any other molecular class. In order to discover if any traditional medicine or other compounds could reduce morbidity and mortality from SBE, a pharmaceutical drug discovery approach is required. The test compound needs to be specific, i.e., inhibit the action of the venom toxin(s) with minimal impact on the human proteins. It needs to be potent, such that only a small dose is needed. Typically, an effective dose of below 50 micromolars is required. To make a major difference to patients, good oral bioavailability and stability on the shelf in tropical temperatures is critical. Finally, the cost of production should be cheap such that the final market price of the treatment is affordable for the communities that need it most. Due to adverse side effects associated with the antivenom therapy in some patients, there is no doubt that we need to develop alternative treatments to overcome these issues. However, as with any safety protocol, prevention of any adverse effects should be prioritised over treatment. With SBE, education is key, helping communities understand the direct connection with their actions and the risk of envenomation. Logical actions such as avoiding attracting rodents (major snake prey) and wearing protective footwear have a major impact in reducing SBE events, but there can be a mismatch between understanding and actions [39].

**Figure 1:**
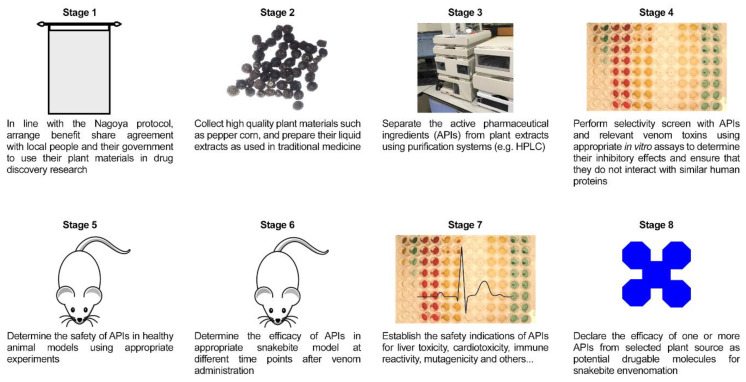
Outline of the suggested process to follow before claiming a potential compound or mixture has potential as a therapeutic for SBE.

Like viper venoms, hepatitis C has evolved a serine protease that is critical in its mode of action. Therefore, using the example of the discovery of a clinically validated viral protease inhibitor, we can hypothesise a similar approach for venom protease inhibitors. From a lead starting point of a hexapeptide derivative with an 800 μM IC_50_, optimisation by Boehringer Ingelheim led to a compound with an IC_50_ of 11 nM [40]. This compound was then tested against human serine proteases, where it had no effect up to 30 μM [40]. This difference between the target efficacy and the off-target effects is called the therapeutic window, and in this case, it was ≈2700 fold. The next step was to demonstrate metabolic stability and oral bioavailability. With a further tweak, the lead compound had an IC_50_ of 6.4 nM, and an oral dose of 20 mg/kg led to a plasma concentration of 2.5 μM [40]. This is more than sufficient for full target engagement, but not enough to inhibit the human proteins. This compound went on to reduce the viral load in human patients. The process for getting a plant extract to a point where it can legitimately be said that it could be a potentially effective treatment for SBE is outlined in Figure 1.

## 8. Conclusions

Venoms are far more complex than viral genomes. However, it is common knowledge in the field that the majority of the pathologies from a particular species are often down to just a few gene/toxin families. A systematic approach is within our grasp to take venom enzyme inhibitors previously identified from plants and other sources, through to identifying the specific API(s) and actually benefitting SBE patients. Finding a lead series of specific potent inhibitors with selectivity over human enzyme orthologues will benefit SBE patients. They still need to be screened for oral bioavailability, as well as thermal and serum stability, but this will lead to datasets that can be labelled as “potential cures for SBE” if they can get to the site of action when dosed at a reasonable timepoint post-bite. This can all be achieved if less effort is put into trying to replicate the WHO test for antivenoms with compounds that are not antibodies and more effort is focused on the drug discovery process. There may well be excellent compounds in the ethnobotanical pharmacopeia, but the majority of studies have not been robust enough to identify them. Investigating such leads should also include protecting the natural resources, as well as the knowledge around traditional medicine, in line with the convention on biodiversity and the Nagoya protocol on access and benefit sharing [41]. Once the APIs are identified and characterised from the plant sources, they can then be chemically synthesised in order to conserve the plants while producing the APIs in large quantities to meet the demand.
